# The marketing of commercial foods for infants and toddlers in Australian supermarket catalogues

**DOI:** 10.1093/heapro/daaf043

**Published:** 2025-06-11

**Authors:** Alexandra Chung, Sophia Torkel, Helen Dixon, Jennifer McCann, Andrea Schmidtke, Catharine Fleming

**Affiliations:** Department of Nutrition, Dietetics and Food, Monash University, 1/264 Ferntree Gully Rd, Notting Hill, Victoria, 3168, Australia; Faculty of Medicine, Nursing, and Health Sciences, Monash Centre for Health Research and Implementation (MCHRI), Monash University, Level 1, 43-51 Kanooka Grove, Clayton, Victoria, 3168, Australia; Centre for Behavioural Research in Cancer, Cancer Council Victoria, Level 8, 200 Victoria Parade, East Melbourne, Victoria, 3002, Australia; Melbourne School of Psychological Sciences, The University of Melbourne, 12/17 Spencer Road, Parkville, Victoria, 3010, Australia; Institute for Physical Activity and Nutrition (IPAN), School of Exercise and Nutrition Sciences, Deakin University, 221 Burwood Highway, Burwood, Victoria, 3125, Australia; Food for Health Alliance, Level 8, 200 Victoria Parade, East Melbourne, Victoria, 3002, Australia; School of Health Science, Western Sydney University, Narellan Road and Gilchrist Drive, Campbelltown, New South Wales, 2560, Australia

**Keywords:** early childhood, diet, nutrition, commercial determinants of health

## Abstract

Commercial foods for infants and young children are prominent on supermarket shelves in Australia, with parents commonly believing they are a healthy choice, yet evidence shows many commercial foods are nutrient-poor. The aim of this study was to examine the nature and extent of promotions for commercial infant and toddler foods in Australian supermarket catalogues. Digital catalogues from four leading Australian supermarket chains were collected and content analysed over 12 weeks from August to October 2023 (*n* = 60 catalogues with 2206 pages). Using a coding guide, one researcher coded all advertised products to identify commercial infant and toddler foods and recorded the labelled age range, product category, packaging type, and associated promotions for each product. A total of 121 commercial infant and toddler food products were identified across 49 catalogue pages (3.5% of all pages examined). The most advertised categories of commercial infant and toddler foods were fruit purees (40%), snacks (27%), and confectionary (12%); 74% of advertised commercial foods were labelled for infants under 12 months; 26% were labelled for toddlers 12–36 months of age; and 50% of products were packaged in pouches. Techniques used to promote commercial infant and toddler foods included price (95%) and health-related messaging (20%). Foods promoted for infants and young children in Australian supermarket catalogues are misaligned with the recommendations within Australia’s Infant Feeding Guidelines. There is an urgent need to reduce the promotion of packaged commercial infant and toddler foods in supermarket catalogues to better support and promote healthy diets for young children.

Contribution to Health Promotion

- Commercial foods for infants and toddlers are nutritionally poor and frequently display on-pack marketing that is incongruent with infant and toddler feeding guidelines.
- Supermarket catalogues in Australia predominantly promote packaged commercial foods for infants and toddlers using price promotions and health-related messaging.
- Restricting the promotion of commercial foods for infants and toddlers in supermarket catalogues should form an important part of comprehensive policy efforts to curtail the unhealthy influence of food marketing on children’s diets and reorient this powerful marketing medium towards promoting healthier whole foods over processed, packaged commercial foods for infants and toddlers.

## BACKGROUND

Unhealthy diets such as those low in fruit, vegetables, legumes, and whole grains and/or high in excess fat, sugar, and sodium are a leading cause of the burden of disease (Stanaway et al. 2018). As children’s dietary patterns are developed in infancy and persist throughout childhood (EDEN Mother–Child Cohort Study Group 2015, Luque et al. 2018), establishing healthy eating habits early in life is crucial for lifelong health (World Health Organization 2014). Australia’s Infant Feeding Guidelines (AIFGs) recommend that infants are exclusively breastfed until 6 months of age, after which time solid foods can be introduced alongside continued breastfeeding (National Health and Medical Research Council 2012). First foods should be iron-rich, nutritious and appropriate in texture for the developmental age of the child. Juices, sugary drinks, and foods with added sugars, salt or high levels of saturated fat should be avoided (National Health and Medical Research Council 2012). From 12 months of age and beyond, toddlers (aged 12–36 months), should eat family foods consistent with the Australian Dietary Guidelines (ADGs) which recommend consuming a wide variety of foods from five food groups (vegetables/legumes, fruits, grains, meat/alternatives, and dairy/alternatives; collectively referred to as core foods) and limiting the intake of foods containing saturated fat, salt, and sugars (discretionary foods) (National Health and Medical Research Council 2013). Concerningly, many Australian infants’ and toddlers’ diets fall short of these recommendations. For example, 80% of Australian children aged 2–3 years do not meet the recommended intake of fruit and vegetables each day (Australian Bureau of Statistics 2022). Among young children in Australia, more than 60% regularly consume unhealthy snack foods and sweet drinks (Chung et al. 2018) with discretionary foods accounting for 30% of total energy intake among 2- to 3-year-olds (Australian Institute of Health and Welfare 2018).

It is well established that food marketing has an influential role in children’s diets in Australia and internationally (Smith et al. 2019, Boyland et al. 2022). Marketing of food and beverages influences children’s dietary behaviours, increases their preference, choice, and consumption of advertised products (Boyland et al. 2022) and influences parents’ product perceptions and food choices (Chung et al. 2024). Food marketing is ubiquitous, on television and digital media, in public spaces, and throughout retail environments including in-store promotions, on food packaging, and via supermarket catalogues distributed in hardcopy and online (Smith et al. 2019, Tan et al. 2023).

Food marketing disproportionately promotes unhealthy, discretionary, foods over core foods such as cereals and wholegrains, vegetables and legumes, fruit, dairy, meat and alternatives (World Health Organization 2022), and the Australian retail environment is no exception. Australian supermarket shelf space and check-out displays are more likely to promote discretionary products than core products (Schultz et al. 2021). In-store price promotions are also more likely to promote discretionary products (Grigsby-Duffy et al. 2020) and Australian supermarket catalogues promote more discretionary food and beverages than core food products (Cameron et al. 2017). The result is a food marketing environment that promotes unhealthy products at multiple points along a consumer’s ‘path to purchase’ (Moran et al. 2022), nudging consumers towards unhealthy eating behaviours.

Commercial infant and toddler foods (CITFs) include manufactured foods or drinks (other than breastmilk substitutes) which are marketed as suitable for feeding infants (<12 months old) and toddlers (12–36 months old) (World Health Organization 2023). In Australia, under the Food Standards Code, there is some regulation of foods for infants, but very limited regulation of the promotion of foods for toddlers. These products represent a rapidly expanding category in the Australian grocery market, led by the recent growth of products targeted towards toddlers, which predominantly comprise ultra-processed snack foods (McCann et al. 2021a). Commercial squeeze pouch products have also experienced significant market growth and now dominate commercial infant and toddler food markets globally estimated to be worth US$2111 million in 2018 and spout pouch packaging is expected to drive global markets at 7.5% over 2018–26 (Transparency Market Research, 30 April 2018). CITFs are strategically marketed through a range of media and settings to reach consumers at various touchpoints along the path to purchase. These products and their extensive promotion are designed to appeal to parents and carers as well as young children (Chung et al. 2023). To date, most research on CITFs has examined the composition, labelling, and packaging of the products themselves (McCann et al. 2021a, 2021b, Chung et al. 2023), but little is known about the extent and manner in which these products are promoted via other influential marketing channels such as supermarket catalogues.

Australian research has found CITFs, including snacks and squeeze pouches, are nutritionally poor, high in sugar, and frequently display on-pack promotional messages and labels that are incongruent with infant and toddler feeding guidelines (McCann et al. 2021a, Brunacci et al. 2023, Chung et al. 2023, Scully et al. 2023). Recently, the World Health Organization Regional Office for Europe published the Nutrient and Promotion Profile Model: supporting the appropriate promotion of food products for infants and young children 6–36 months in the WHO European Region (NPPM) (World Health Organization 2023). Assessments of CITFs on the Australian market have found that a majority of products do not meet the NPPM recommendations for nutrient composition, and no products meet the recommendations for labelling or promotion (Chung et al. 2024, Scully et al. 2024). Similar findings have been reported in assessments of CITFs in the USA (Coyle et al. 2024) whilst examination of CITFs across several countries in South East Asia found evidence of poor nutrient composition including high sugar content, inappropriate product labelling, and extensive use of promotional claims (Bassetti et al. 2023).

CITFs are frequently purchased by parents and carers for their young children, with one in two parents in an Australian survey reporting that these foods made up at least half of their child’s overall dietary intake (The Royal Children’s Hospital Melbourne 2022). Reasons for choosing CITFs include convenience, children liking the taste, and parents’ perceptions that the products are healthy and nutritious, and affordable or inexpensive (The Royal Children’s Hospital Melbourne 2022, Walls et al. 2023, Hollinrake et al. 2024). Parental anxiety and uncertainty around food preparation and concerns about food safety also lead parents to choose CITF for their children (Isaacs, Neve et al. 2022, Neve, Coleman et al. 2024). Sole parents and families experiencing socioeconomic disadvantage are more likely to choose CITFs to feed their young children (The Royal Children’s Hospital Melbourne 2022, Neve, Coleman et al. 2024). Studies assessing parents’ and carers’ responses to front-of-pack claims on CITFs have found that claims highlighting desirable product attributes (e.g. ‘preservative free’) can confer a ‘health halo’ on otherwise unhealthy products, boosting perceptions of the healthiness and suitability of these products for young children, as well as increasing preferences and purchasing intentions, with certain parent subgroups (e.g. those with three or more children) showing greater responsiveness to such claims (McCann et al. 2022, Chung et al. 2024, Dixon et al. 2024). Together these findings suggest that the manner in which CITFs are marketed to parents warrants wider scrutiny.

Given the importance of early childhood feeding for lifelong health (Mameli et al. 2016, Moore et al. 2017, World Health Organization 2021), and the role of food environments in shaping dietary behaviours (Swinburn et al. 2011), there is a need to build a more comprehensive understanding of how CITF products are marketed by food retailers. This evidence is necessary to create food environments that support optimal child health and development. Supermarket catalogues have vast audience reach, capture audience attention longer than other advertising (6 min on average, with 30% reading them cover-to-cover), are considered by consumers to be the most useful media in relation to grocery purchasing, and are known to drive people in the store and trigger unintended purchases (Roy 2017, The Real Media Collective 2018, Roy 2019, Sprinter 2019). Industry research from Australia suggests that 75% of shoppers use catalogues to decide what products to buy, with digital catalogues accessed more frequently than traditional paper catalogues (Rider 2023). Thus, the aim of this study was to assess how infant and toddler feeding is portrayed in Australian supermarket catalogues including the types of products promoted, and the promotional techniques used.

## METHODS

### Data collection

A systematic content analysis was undertaken to identify the extent and manner in which CITF products are represented within supermarket catalogues. Weekly catalogues were downloaded from the websites of Australia’s four major supermarket chains (Aldi, Coles, IGA, and Woolworths) for 12 consecutive weeks (August to October 2023). These four supermarket chains have a majority market share of more than 70% (McCann et al. 2022). The most read catalogues are from Aldi, followed by Woolworths and Coles (The Real Media Collective 2018). In the first week of the study, catalogues from each of the four supermarket chains were downloaded for store locations in the first and fifth quintile of the Index of Relative Socioeconomic Disadvantage across each Australian state and territory. A pre-test comparison found that the Aldi, Coles, and Woolworths catalogues had no differences according to postcode, and only minor differences in seasonal produce, locally sourced items, and pricing between states/territories. Therefore, a single weekly catalogue was downloaded for each of Aldi, Coles, and Woolworths using a central Sydney metropolitan postcode. Heterogeneity in IGA catalogues was observed according to store type, so two IGA catalogues were downloaded each week (one each from IGA and Supa IGA). A pre-test comparison of digital catalogues against a sample of printed catalogues for the same period found only minor formatting variations between digital and hardcopy versions, as such digital catalogues were used throughout the study.

### Data coding and analysis

A coding guide with predefined drop-down responses was developed by the research team and piloted by A.C. and S.T. To ensure the reliability of the coding process, peer verification was conducted whereby the two researchers discussed their coding interpretations first with one another and then with the wider research team. Each digital catalogue page was coded for the presence of food and beverages, with foods and beverages classified as either healthy, discretionary, or CITF. The presence of CITFs was determined by the presence of products displaying a recommended age range between 4 months and 3 years on product packaging. Infant formula and toddler milks were not classified as CITFs in this study.

For each page that displayed CITFs, the page was further analysed to determine the presence and nature of accompanying imagery (e.g. fresh foods, parent or child, serving suggestions, shopping trolley), promotional text (e.g. text denoting health benefits, suitability for babies, sustainability), nearby infant formula or toddler milks, or nearby non-food infant or toddler products (e.g. nappies, baby wipes, or baby skincare products).

For each CITF product displayed, the following data were recorded: recommended age range on product label (infant or toddler); product category based on the NPPM categories (World Health Organization 2023): dry cereals, dairy, fruit puree (containing > 5% fruit), vegetable puree, savoury meal, snacks and finger food (snacks), confectionery (includes chocolates and sweets as well as dried and processed fruit products), drinks, or ingredients; packaging type (pouch, jar, box, or bag); and price including whether the price was listed, whether a discount was promoted and whether the item was promoted as having an everyday low price. All promotions for everyday low prices were coded as such regardless of whether the actual price was listed.

Data were extracted into a Microsoft Excel sheet prepared with predefined drop-down fields and all coding was performed by an Accredited Practising Dietitian (ST). All data analysis was performed in Microsoft Excel. Descriptive statistics were reported as frequencies and relative frequencies, with stratification by supermarket and target age. The Pearson chi-square test was used for significance testing to compare page distribution by supermarket brand, WHO NPPM category by age range and package type by age range, with Pearson residuals calculated for post-hoc testing.

## RESULTS

### Overall catalogue characteristics

**Figure 1.**
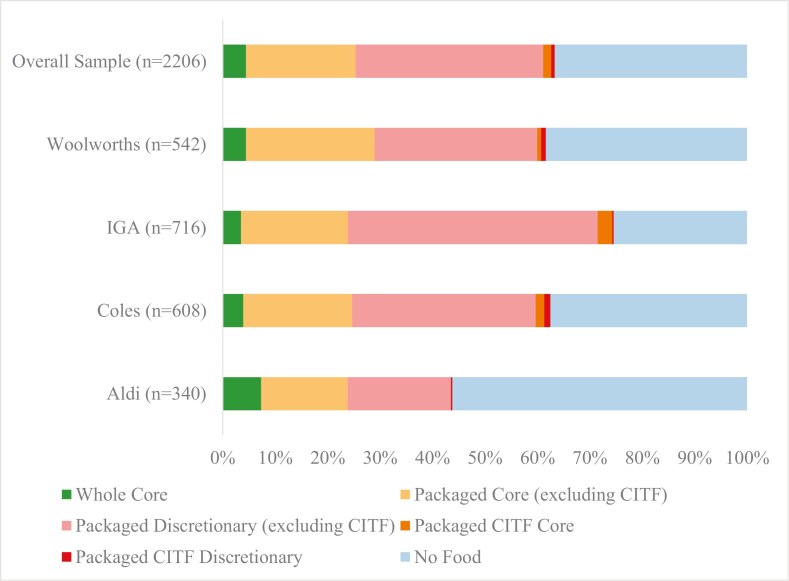
Proportion of catalogue pages allocated to each product category.

Sixty individual catalogues comprising 2206 pages were analysed. Almost two-thirds of all supermarket catalogue pages promoted food and beverages (63%). Of the pages promoting food and beverages, 56% of pages promoted discretionary (unhealthy) food, 33% promoted packaged core (healthy) foods, 7% promoted fresh core (healthy) food, and 3.5% promoted CITFs (Fig. 1). The pages allocated to whole core, packaged core (including CITF), packaged discretionary (including CITF) and non-food items differed significantly between supermarkets (χ^2^ = 130, df = 9, *P* < .00001). Compared to the other supermarket chains, IGA had the highest proportion of catalogue pages promoting packaged discretionary food (Pearson residual = 5.1), whilst Aldi had the highest proportion of catalogue pages promoting non-food products (Pearson residual = 5.9) and the lowest proportion of pages promoting packaged discretionary foods (Pearson residual = −5.0).

### Catalogue promotions of CITFs

Thirty-five catalogues (58%) included promotions for CITFs, depicted across 49 catalogue pages. Coles catalogues most frequently included CITFs, with 92% of catalogues featuring these, whilst 67% of Woolworths catalogues and 63% of IGA catalogues, respectively, included CITFs. Only one Aldi catalogue included CITFs during the study period. IGA catalogues had the highest proportion of pages featuring CITFs (3.1%), followed by Coles (2.8%), Woolworths (1.7%), and Aldi (0.29%).

### Types of CITF promoted in catalogues

Seventy-four percent of all CITFs promoted in catalogues were labelled for consumption by infants (< 12 months), of these 21% were labelled as being suitable for 4 + months. Twenty-six percent of products were labelled as suitable for toddlers (12–36 months).

Fruit purees were the most frequently promoted category of CITFs (40%), followed by snacks (27%), confectionary (12%), savoury meals (7.4%), dry cereals (6.6%), dairy (5%), and vegetable puree (1.7%). The proportion of products in each category labelled for consumption by infants compared to toddlers differed significantly (χ^2^ = 12.6, df = 2, *P* = .002), with fruit purees more commonly labelled for consumption by infants rather than toddlers (Pearson residual = −2.0).

Half of the CITFs promoted were packaged in a pouch (50%), with this form of packaging more prevalent among infants than toddler foods (59% cf. 20%, χ^2^ = 14.0, df = 1, *P* = .0002). For infant foods, pouches were the most common type of packaging, followed by bags (30%) and boxes (11%). For toddler foods, the most common packaging types were boxes (47%), followed by bags (27%) and pouches (20%) (Table 1). The proportion of products packaged in pouches was significantly different between infant foods and toddler foods (χ^2^ = 14.0, df = 1, *P* = .0002).

**Table 1.** Age range, nutritional profile, and packaging characteristics of commercial infant and toddler foods featured in supermarket catalogues overall and by retail chain.

	Overall sample *n* = 121 *n* (%)	Aldi *n* = 8 *n* (%)	Coles *n* = 54 *n* (%)	IGA *n* = 41 *n* (%)	Woolworths *n* = 18 *n* (%)
**Age range**					
Infant (< 12 months)	91 (75%)	2 (25%)	40 (74%)	34 (83%)	15 (83%)
Toddler (12–36 months)	30 (25%)	6 (75%)	14 (26%)	7 (17%)	3 (17%)
**Minimum age (months)**					
4+	26 (21%)	0 (0%)	5 (9.3%)	21 (51%)	0 (0%)
6 + or 7+	33 (27%)	2 (25%)	15 (28%)	8 (20%)	8 (44%)
8 + or 10+	30 (25%)	0 (0%)	20 (37%)	3 (7.3%)	7 (39%)
12+	32 (26%)	6 (75%)	14 (26%)	9 (22%)	3 (17%)
**WHO NPPM category**					
Dry cereals	8 (6.6%)	0 (0%)	1 (1.9%)	5 (12%)	2 (11%)
Dairy	6 (5.0%)	0 (0%)	5 (9.3%)	1 (2.4%)	0 (0%)
Fruit puree	48 (40%)	0 (0%)	17 (31%)	23 (56%)	8 (44%)
Veg puree	2 (1.7%)	0 (0%)	2 (3.7%)	0 (0%)	0 (0%)
Savoury meal	9 (7.4%)	0 (0%)	1 (1.9%)	8 (20%)	0 (0%)
Snacks	33 (27%)	2 (25%)	21 (39%)	2 (4.9%)	8 (44%)
Confectionary	15 (12%)	6 (75%)	7 (13%)	2 (4.9%)	0 (0%)
**Packaging type**					
Bag	35 (29%)	2 (25%)	20 (37%)	4 (9.8%)	9 (50%)
Box	24 (20%)	6 (75%)	10 (19%)	7 (17%)	1 (5.6%)
Jar	2 (1.7%)	0 (0%)	2 (3.7%)	0 (0%)	0 (0%)
Pouch	60 (50%)	0 (0%)	22 (41%)	30 (73%)	8 (44%)

### Techniques used to promote CITF in catalogues

A range of promotional features were used to promote CITFs in supermarket catalogues. Price promotions were most common with 56% of products featuring a price discount, and a further 39% of products advertised with an ‘everyday low price’ slogan. Written text was observed on many of the pages promoting CITFs, including baby-related text (e.g. the word ‘baby’ on the page heading) (55%); health-related text (such as the word ‘health’ on the page heading) (20%); and text about the community (12%) and environment (2%). Visual promotions alongside CITFs included images of core foods (fresh fruit, vegetables, dairy, or grains) (16%); images of babies or children (8.2%); and toys and baby/child-themed props such as alphabet blocks and garlands (10%). CITFs were commonly promoted on the same page as other infant and toddler products such as nappies and wipes (90%). More than half the pages promoted infant formula or toddler milks alongside CITFs (55%) including four promotions for stage 1 or 2 formula and 22 promotions for stage 3 or 4 toddler milk drinks (Table 2).

**Table 2.** Techniques used to promote commercial infant and toddler foods within supermarket catalogues overall and by retail chain.

Page-level techniques	Overall sample *n* = 49 *n* (%)	Aldi *n* = 1 *n* (%)	Coles *n* = 17 *n* (%)	IGA *n* = 22 *n* (%)	Woolworths *n* = 9 *n* (%)
*Adjacent imagery*					
Whole fresh core foods	8 (16%)	0 (0%)	0 (0%)	8 (36%)	0 (0%)
Child/baby	4 (8.2%)	0 (0%)	2 (12%)	2 (9.1%)	0 (0%)
Toys and props	11 (22%)	1 (100%)	3 (18%)	6 (27%)	1 (11%)
Formula milk	27 (55%)	0 (0%)	15 (88%)	8 (36%)	4 (44%)
Non-food baby products	44 (90%)	1 (100%)	17 (100%)	17 (77%)	9 (100%)
*Adjacent text*					
Suitability for babies/toddlers	27 (55%)	0 (0%)	3 (18%)	16 (73%)	8 (89%)
Health benefits	10 (20%)	0 (0%)	0 (0%)	8 (36%)	2 (22%)
Sustainability	1 (2%)	1 (100%)	0 (0%)	0 (0%)	0 (0%)
Community or charity-related	6 (12%)	0 (0%)	0 (0%)	6 (27%)	0 (0%)

Product-level techniques	Overall sample *n* = 121 *n* (%)	Aldi *n* = 8 *n* (%)	Coles *n* = 54 *n* (%)	IGA *n* = 41 *n* (%)	Woolworths *n* = 18 *n* (%)
*Any price promotions (Special price promotion or everyday low price)*	115 (95%)	8 (100%)	54 (100%)	35 (85%)	18 (100%)
Price listed	109 (90%)	8 (100%)	54 (100%)	29 (71%)	18 (100%)
Special price promoted	68 (56%)	0 (0%)	54 (100%)	5 (12%)	9 (50%)
Everyday low price	47 (39%)	8 (100%)	0 (0%)	30 (73%)	9 (50%)

## DISCUSSION

This study presents a novel objective assessment of the promotion CITFs in a sample of 60 Australian supermarket catalogues over a period of 12 weeks. Packaged CITFs were observed in more than half of the catalogues examined in this study, and half of the CITFs were packaged in a pouch. Fruit purees and snacks were the most promoted categories CITFs in supermarket catalogues, echoing the predominance of these kinds of CITF products in online supermarkets and in-store (Scully et al. 2023), posing concerns for infant and toddler nutrition.

Commercial food pouches in Australia are high in sugar, lack necessary iron, and can impede the development of oral motor skills and eating behaviours if consumed directly from the spout (Brunacci et al. 2023). It is therefore concerning that current findings demonstrated that fruit puree pouch products along with snacks are the most promoted CIFTs in Australian supermarket catalogues. When choosing infant and toddler foods, convenience is a key consideration among parents in Australia (The Royal Children’s Hospital National Child Health Poll 2022, Chung et al. 2023) and the UK (Hollinrake et al. 2024, Neve et al. 2024). The prominence within catalogue promotions of CITFs packaged in ways that babies and toddlers can self-feed with minimal mess (e.g. pouches or hand-held snacks) demonstrates that industry is capitalizing on parent concerns around convenience when feeding their young children. However, the problem with these ‘convenient’ types of CITFs being heavily promoted to parents is that many of them contain fruit purees which are a concentrated source of free sugar, contributing significant energy and increasing the risk of dental caries and excessive energy intake for young children who consume pouches frequently (Brunacci et al. 2023). In addition, it is well established that the first foods and flavours infants are exposed to form the basis of eating patterns and food preferences into adulthood (Ventura and Worobey 2013, De Cosmi et al. 2017). If infants regularly consume very sweet fruit-based purees with limited exposure to bitter or vegetable-based foods, they may acquire a taste preference for sweet foods, which can result in eating patterns associated with negative health outcomes (Ventura and Worobey 2013, De Cosmi et al. 2017).

CITF snack foods are often ultra-processed and often of poorer nutritional quality than similar foods found in other parts of the supermarket (McCann et al. 2021a, 2022). Snacking is common among young children, and packaged snacks appeal to parents and caregivers, despite their poor nutritional value (Coleman et al. 2023). The promotion of packaged snacks in supermarket catalogues and within retail settings implicitly perpetuates the culture of snacking among infants and toddlers, which may serve to normalize the consumption of ultra-processed snack foods in place of healthier, more nutritious foods in the diet. Frequent consumption of packaged discretionary food among young children is of public health concern. Australia’s Infant Feeding Guidelines recommend young children consume a variety of foods from five food groups (vegetables/legumes, fruits, grains, meat/alternatives, and dairy alternatives; collectively referred to as core foods) and limited intake of foods containing saturated fat, added salt, and added sugars. They also explicitly state that special complementary foods or milks for toddlers are not required for healthy children (National Health and Medical Research Council 2012). Displacing core foods that provide nutrients necessary for growth and health such as iron, can place a child at risk of micronutrient deficiencies, impacting their growth and health outcomes (National Health and Medical Research Council 2013).

Theory and research draw attention to the importance of visual communication as well as text-based communication in relation to food packaging (Kelly et al. 2024). The present study applied this reasoning to examining CITF promotions within supermarket catalogues by assessing the text and imagery employed in this form of advertising. Nutrition-related text and images of healthy, whole foods were common techniques used to promote CITF throughout supermarket catalogues. Similarly, nutrition-related claims are commonly used on the front-of-pack of foods for infants and toddlers (Chung et al. 2023). Research has shown that front-of-pack nutrition-content claims increase parents’ perceptions of healthiness of ultra-processed, discretionary toddler snack foods and ultra-processed toddler milks (McCann et al. 2022). ‘Free from bad ingredient’ claims are particularly influential in boosting parents’ perceptions of and purchase intentions towards unhealthy toddler snack foods (Dixon et al. 2024). This phenomenon is the result of a cognitive bias known as the ‘health halo effect’, whereby claims highlighting isolated positive nutrition attributes of products lead consumers to generalize about a product’s overall nutritional profile (Choi et al. 2013, Mediano Stoltze et al. 2021). This persuasive technique is also being harnessed within supermarket catalogue promotions for CITF. In regards to the depiction of healthy whole foods alongside processed CITF within supermarket catalogues, recent research found that the inclusion of images of whole foods on packaged lunchbox snacks influenced parents to perceive these products as healthier and increased their intentions to purchase these products for their children (Dixon et al. 2024). It seems that pairing processed packaged foods with images of healthy whole foods leads parents to perceive these foods as more healthful.

Over half the catalogues featured text that mentioned babies or toddlers on the pages that displayed CITFs. Child-related claims such as ‘for little hands’ are also common on CITF packaging (Garcia et al. 2022, Chung et al. 2023); recent research found these claims increased intentions to purchase these products among sole parents (but not other parents) (Dixon et al. 2024), a population known to be especially reliant on CITFs for feeding their babies and toddlers (The Royal Children’s Hospital Melbourne 2022). With concerns surrounding the frequent use of commercial products such as squeeze pouches beyond the early complementary feeding period (6–7 months) due to possible impact on long-term feeding behaviours and dietary intake in young children, promoting squeeze pouch products to vulnerable parents can have long-term impact on feeding development. By 12 months of age the child should be consuming a wide variety of family foods (NHMRC 2012) and not require the smooth puree textured foods found in commercial squeeze pouch products. Extended use of puréed foods and a delayed introduction of lumpy textures beyond the age of 9 months has been associated with feeding difficulties in older children and a lower intake of vegetables and fruit (Koletzko et al. 2019).

Prevalent depictions within media content are predicted to influence ‘real world’ perceptions and behaviour among audiences with heavy exposure to that content (Gerbner et al. 2022). CITFs were commonly placed alongside other ‘baby’ products in catalogues including infant and follow-on formula, nappies and baby wipes. This explicit placement allows us to infer that parents of babies and toddlers are the target audience for these sections of supermarket catalogues, with the subtext that all baby needs can be met in a ‘one-stop shop’. This implicit funnelling of parent’s purchasing for their little ones towards packaged products (and away from whole food) persists at the point-of-sale, both in-store (dedicated baby product aisle or section) and online (virtual supermarkets typically have a ‘baby’ section) where the only food products displayed are CITFs, not whole foods. In this manner, the promotion of CITFs may serve to normalize these products as the most appropriate foods for babies and toddlers.

Furthermore, the placement of CITF alongside infant formula implies an association between these products that undermines breastfeeding (Laws et al. 2022). As outlined by WHO and in AIFGs, complementary foods are designed to supplement breastfeeding from 6 months of age up to and beyond 2 years of age (National Health and Medical Research Council 2012, World Health Organization Regional Office for Europe 2019). Of particular concern, more than one-fifth of the CITF products promoted in supermarket catalogues were labelled as being suitable for infants from 4 months of age. This labelling contravenes WHO breastfeeding recommendations and the AIFGs which both recommend exclusive breastfeeding until 6 months, with the introduction of solid foods from around 6 months (National Health and Medical Research Council 2012, World Health Organization Regional Office for Europe 2019). The promotion of products as suitable from 4 months also breaches the WHO CODEX, which states ‘*complementary foods should not carry messages or contain information which may lead mothers and caregivers to believe that these products are suitable for infants below 6 months of age*’ (World Health Organization 2017). The promotion of CITFs marketed from 4 months of age (16) coupled with the promotion in close proximity to infant and follow-on formula in supermarket catalogues, there is a risk of undermining and displacing breastfeeding during this critical early life stage (Brunacci et al. 2023).

This study also found that price promotions were frequently used to promote CITFs within supermarket catalogues. Price promotions are a powerful strategy for influencing food purchase decisions (Schultz et al. 2021) and are more common for unhealthy food and beverages (Blake et al. 2021). Furthermore, price strategies that encourage the purchase of healthier products may help to reduce the socioeconomic gradient in health, given that people with lower incomes are more sensitive to food prices (Mackenbach et al. 2019). Previous survey findings revealing that sole parents and parents with lower levels of education are more reliant on CITFs (The Royal Children’s Hospital Melbourne 2022), point to the possibility that price-related promotions on these products may be contributing to this phenomenon.

It is notable that within the catalogues examined in this study, a minority of pages (2.2%) featured promotions of packaged CITFs, and CITFs containing core foods were slightly better represented than discretionary CITFs. Nonetheless, given the vast audience reach and impact on purchasing of these catalogues (Roy 2017, The Real Media Collective 2018, Roy 2019, Sprinter 2019), as well as the dominance of packaged products rather than whole foods within sections of the catalogue that were clearly targeted at parents of babies and toddlers, this is concerning as these catalogue promotions may serve to normalize feeding babies and toddlers packaged foods rather than healthier meals and snacks prepared from whole foods. An Australian audit of supermarket catalogues conducted in 2013 found discretionary foods comprised 43% of the total catalogue whilst core foods such as fruit, vegetables, legumes, and nuts comprised just 10% of the catalogue content (Cameron et al. 2017). Similar findings have also been observed internationally. For example, a 2015 audit of Dutch supermarket catalogues observed most promotions were for unhealthy products with 70% of promotions categorized as unhealthy (Ravensbergen et al. 2015). These findings demonstrate the ways in which the retail food sector contributes to the promotion of unhealthy diets.

The retail food environment offers a key opportunity for action to support healthy childhood diets (Blake et al. 2021). Interventions to improve the supermarket food environment have been shown to improve the healthiness of consumer purchases (Cameron et al. 2016).

Public health experts agree that policies that reduce the influence of unhealthy food and drink promotions are an important component in a comprehensive approach to improving population diets (Backholer et al. 2019, McCann et al. 2024). This research demonstrates the need for greater promotion of healthy food, especially fresh food, and reduced promotion of packaged and unhealthy food in supermarket catalogues, particularly for infants and toddlers.

### Strengths and limitations

Content analysis offers a replicable and reliable tool for documenting patterns in recorded communications, to identify recurring messages and themes. However, it is subject to limitations. Content analysis may involve subjectivity on the part of the coder; inferences about message impact cannot be made based on content analysis alone; and reductive coding to pre-determined categories can obscure nuanced messaging. Furthermore, our focus on digital catalogues for the four supermarket chains occupying 70% of the market share in Australia means we did not examine the promotion of CITFs by smaller, independent retailers. Nonetheless, the present study helps address a gap in the literature regarding the ways CITFs are promoted within catalogues by major Australian supermarket retailers. The association between the promotion of CITFs (McCann et al. 2021a, 2021b, Chung et al. 2023) and parent’s perceptions of these products (McCann et al. 2022, Chung et al. 2024, Dixon et al. 2024) suggest that industry marketing for CITFs may be both appealing to and driving parent’s perceptions and purchasing preferences for CITFs. Future research should explore parents’ responses to CITF promotions within supermarket catalogues (as well as across other media and settings), including experimental research where the direction of causality of marketing impacts can be established to expand our understanding of how CITF promotion influences parents’ beliefs and behaviours with regards to their children’s diets. Routine monitoring of the techniques used to promote CITFs in supermarket catalogues at different timepoints throughout the year would also help to provide a comprehensive picture of variations and trends in marketing strategies. In jurisdictions where changes to regulation around the promotion of CITF occur, it will be important to monitor how these changes impact industry practices and consumer responses.

## CONCLUSION

Food choices are influenced by the interplay between biological, economic, physical, social, and psychological factors. In the case of infant and toddler feeding, there is a unique parent–child consumer dyad, whereby the impact of the food marketing environment on parents has implications for their children’s diet and health. Currently, the promotion of infant and toddler foods in Australian supermarket catalogues explicitly encourages purchase and consumption of CITFs, and may implicitly enculturate parents’ perceptions of what constitutes a normal diet for infants and toddlers. The CITFs displayed in the supermarket catalogues in this study were predominantly packaged, processed, foods with a sweet taste, promoted using a range of written, visual, and pricing techniques. The current promotion of CITFs in supermarket catalogues does not support optimal or recommended infant and toddler feeding practices. As supermarket catalogues achieve vast audience reach and engagement and are known to influence grocery purchasing, they represent an important opportunity for interventions to improve the health of the retail food environment and better support healthy infant and toddler diets. Comprehensive government-led regulation that includes restrictions on the promotion of CITFs in supermarket catalogues would create an important lever to curtail the harmful influence of unhealthy food marketing on young children’s diets in Australia.

## Data Availability

The data underlying this article will be shared upon reasonable request to the corresponding author.
